# Machine learning–based prediction of excessive daytime sleepiness in patients with Parkinson’s disease: findings from the PPMI cohort with external validation

**DOI:** 10.3389/fmed.2026.1894008

**Published:** 2026-07-20

**Authors:** Min Li, An Xu, Xiaoguang Luo

**Affiliations:** 1 Department of Neurology, The Second Clinical Medical College of Jinan University, Shenzhen, Guangdong, China; 2 Department of Neurology, Shenzhen People's Hospital (The First Affiliated Hospital, Southern University of Science and Technology, The Second Clinical Medical College of Jinan University), Shenzhen, Guangdong, China; 3 Department of Respiratory and Critical Care Medicine, Shenzhen Institute of Respiratory Diseases, Shenzhen People’s Hospital (The First Affiliated Hospital, Southern University of Science and Technology, The Second Clinical Medical College of Jinan University), Shenzhen, Guangdong, China; 4 Guangdong Provincial Clinical Research Center for Geriatrics, Shenzhen Clinical Research Center for Geriatrics, Shenzhen, Guangdong, China

**Keywords:** external validation, machine learning, Parkinson’s disease, prediction model, sleep disturbance

## Abstract

**Background:**

Excessive daytime sleepiness (EDS) is a common non-motor symptom in Parkinson’s disease (PD). Predicting EDS risk enables early intervention. This study aimed to develop and externally validate an explainable machine learning model for EDS prediction in PD.

**Methods:**

A total of 676 patients with Hoehn and Yahr (H&Y) stage 1 PD from the Parkinson’s Progression Markers Initiative (PPMI) were retrospectively included as the development cohort, randomly split into training and internal validation sets (7:3). An external validation cohort comprised 180 H&Y stage 1 patients from our clinical center. Least absolute shrinkage and selection operator (LASSO) regression and Boruta feature selection identified predictive variables. Four machine learning models were compared, and the optimal model was interpreted using Shapley Additive exPlanations (SHAP).

**Results:**

Five features were selected: Geriatric Depression Scale (GDS), State–Trait Anxiety Index (STAI), Scales for Outcomes in Parkinson’s Disease-Autonomic (SCOPA), Unified Parkinson’s Disease Rating Scale parts I (UPDRS I) and II (UPDRS II). The logistic regression model achieved the best performance, with an area under the receiver operating characteristic curve (AUC) of 0.732 (95% CI: 0.641–0.823) in the internal validation set and 0.673 (95% CI: 0.552–0.793) in the external validation set. SHAP analysis identified key contributors.

**Conclusion:**

We developed and externally validated an explainable machine learning model for EDS prediction in H&Y stage 1 PD. This tool may facilitate early risk stratification and personalized management of sleep problems in this subgroup.

## 1 Introduction

Parkinson’s disease (PD) is a progressive neurodegenerative disorder characterized not only by motor dysfunction but also by a broad spectrum of non-motor symptoms (1). Among these, sleep disturbance is particularly common and clinically important. Sleep-related complaints in PD may include insomnia, difficulty maintaining sleep, excessive daytime sleepiness (EDS), and other manifestations that substantially impair quality of life (1, 2). Increasing evidence suggests that sleep dysfunction in PD is associated with worse emotional status, greater caregiver burden, and poorer functional outcomes (3, 4).

EDS in PD often manifests as an overwhelming and inappropriate propensity to fall asleep during daytime activities, which can severely impair quality of life, increase the risk of accidents, and contribute to caregiver burden (3, 5). Epidemiological studies consistently demonstrate that EDS is a highly prevalent non-motor symptom in PD, with pooled prevalence estimates approximately 50% and increasing with disease progression (6–8). However, its etiology is multifaceted, arising from the neurodegenerative processes intrinsic to PD that disrupt key neural circuits governing sleep–wake regulation, particularly those involving the brainstem, hypothalamus, and basal ganglia (9–11).

The management of EDS in PD requires a personalized, stepwise strategy that begins with a thorough evaluation of potential reversible contributors. Optimization of nocturnal sleep hygiene and treatment of comorbid sleep disorders form the foundational step. Pharmacological review is paramount; this may involve simplifying the regimen, reducing or discontinuing sedating medications (especially dopamine agonists, anticholinergics, or certain antidepressants), or adjusting the timing and dosage of dopaminergic drugs (1, 12–14).

Although EDS is often considered more prominent in advanced PD, clinically significant sleep impairment may already be present in earlier disease stages. Patients with Hoehn and Yahr (H&Y) stage 1 PD are of particular interest because they represent the earliest clinically recognizable stage of PD, with unilateral motor involvement and preserved postural stability. At this stage, non-motor symptoms may emerge and accumulate despite relatively preserved global motor function. Therefore, identifying patients at high risk for EDS during H&Y stage 1 may provide an opportunity for earlier intervention and more individualized management (15, 16).

Previous studies have reported associations between EDS in PD and a variety of factors, including age, disease duration, mood symptoms, anxiety, cognitive impairment, and motor severity. However, most of these studies have focused on prevalence or correlation analyses rather than individualized risk prediction (6, 15). In addition, many previous investigations have included mixed-stage PD populations, which may obscure stage-specific patterns and limit clinical applicability to more homogeneous subgroups (15).

Prediction modeling has emerged as an important strategy for translating routinely collected clinical information into individualized risk stratification. However, EDS in PD is likely driven by complex interactions among neuropsychiatric, autonomic, motor, and treatment-related variables. Machine learning frameworks provide an opportunity to systematically evaluate predictive algorithms with different assumptions and complexities, including both interpretable linear models and more complex nonlinear approaches. Such approaches may facilitate the identification of clinically relevant patterns and support individualized risk assessment (15).

Recent studies have demonstrated the potential of machine learning approaches for PD assessment. For example, Hassan and Ahmed developed a voice-derived prediction framework for monitoring PD progression, highlighting the potential role of data-driven methods in individualized PD assessment (17). Nevertheless, explainable prediction models specifically designed for EDS risk stratification in early-stage PD remain limited.

Prediction studies using machine learning in PD often face two major challenges. First, some models lack external validation, limiting confidence in their generalizability. Second, complex machine learning models are frequently criticized for limited interpretability, which can reduce clinician acceptance and hinder clinical translation. Therefore, integrating external validation and explainable artificial intelligence is important for developing clinically applicable prediction frameworks (15).

In the present study, we adopted a comprehensive predictive modeling framework integrating statistical analysis, comparison of multiple predictive algorithms, external validation, and interpretability analysis. Specifically, we first explored clinical variables associated with EDS, then developed and compared several predictive models with different levels of complexity, externally validated the best-performing model in an independent clinical cohort, and finally applied Shapley Additive Explanations (SHAP) analysis to enhance model transparency. Through this approach, we aimed to construct a clinically applicable and explainable model for EDS risk stratification in patients with H&Y stage 1 PD.

## 2 Methods

### 2.1 Study design and participants

The data used in the study consisted of two cohorts, namely a discovery cohort and an external validation cohort. The discovery cohort was obtained from the Parkinson Progression Markers Initiative (PPMI) cohort (18). The PPMI is an international, multicenter cohort study to identify biomarkers for the progression of PD. We downloaded all the information required for this study from the PPMI database in February 2026.

The original analytic dataset comprised 5,384 individuals with sporadic PD. Inclusion criteria were: (1) age ≥ 30 at diagnosis, (2) drug-naïve or only levodopa preparation, (3) H&Y stage 1 at baseline, (4) disease duration ≤ 2 years, (5) ESS assessment at baseline, (6) PD was diagnosed according to the UK Parkinson’s Disease Society Brain Bank clinical diagnostic criteria (19) and partially confirmed by striatal dopaminergic deficits on single-photon emission computed tomography (SPECT), and (7) the presence of at least two of the following symptoms: resting tremor, bradykinesia, rigidity (mandatory resting tremor or bradykinesia), or asymmetric features. Finally, 676 patients with H&Y stage 1 PD were included in the analysis.

**Figure 1:**
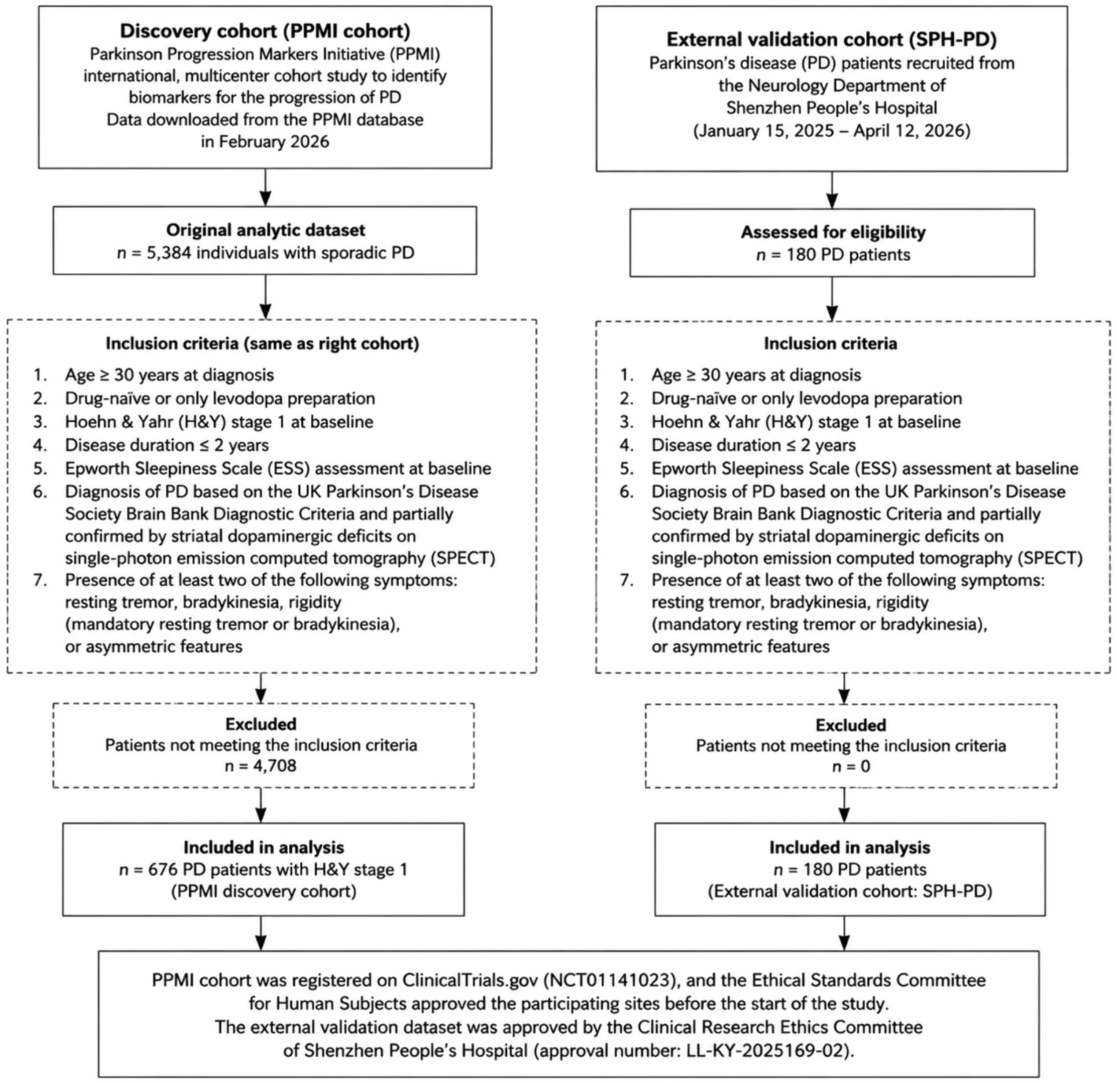
Flowchart of participant selection.

For the external validation cohort, a total of 180 PD patients were recruited who visited the Neurology Department of the Shenzhen People’s Hospital (SPH-PD) from January 15, 2025, to April 12, 2026. The inclusion and exclusion criteria were the same as for the PPMI cohort. The PPMI cohort was registered on ClinicalTrials.gov (NCT01141023), and the Ethical Standards Committee for Human Subjects approved the participating sites before the start of the study. Written informed consent was obtained from all study participants (Figure 1).

### 2.2 Ethics statement

The external validation dataset was approved by the Clinical Research Ethics Committee of Shenzhen People’s Hospital (approval number: LL-KY-2025169-02) and strictly adhered to the ethical principles outlined in the Declaration of Helsinki. All participants gave informed consent.

### 2.3 Measurements

#### 2.3.1 Definition of PD

PD symptoms were assessed according to the Unified Parkinson’s Disease Rating Scale (UPDRS) (20) in the OFF state. The UPDRS consists of four parts evaluating PD patients’ non-motor experiences of daily living, motor experiences of daily living, motor symptoms, and motor complications.

#### 2.3.2 Excessive daytime sleepiness assessment

The Epworth Sleepiness Scale (ESS) is a questionnaire that subjectively measures the daytime sleepiness in adults. Participants have to rate eight questions on a rate of 0–3, with a maximum score of 24. In this study, EDS subjects were participants with an ESS of 10 or higher (21). The ESS with a cut-off point >10 has a 93.5% sensitivity and 100% specificity for distinguishing narcoleptics from the normal population (22).

#### 2.3.3 Covariates

The covariates included in the analysis were age, sex, education level, handedness, body mass index (BMI), disease duration (from PD diagnosis to enrollment, years), dominant side of motor symptoms (DOMSIDE), PD treatment status (PDTRTMNT), levodopa dose, mild cognitive impairment (MCI), Geriatric Depression Scale (GDS), State–Trait Anxiety Index (STAI), Scales for Outcomes in Parkinson’s Disease-Autonomic (SCOPA), UPDRS I, UPDRS II, UPDRS III, ESS. Education level was recorded as the actual number of years, with values capped at 20 years. Handedness was determined using the PPMI standard assessment and classified into three groups: right-handed (RH), left-handed (LH), and mixed-handed (MH). Sex was classified as male or female. BMI was calculated as weight (kg) divided by height squared (m^2^). Participant were classified as having MCI if any 2 or more of the following cognitive tests are >1.5 SD below the standardized mean: (1) Hopkins verbal learning test-total recall (HVLT-Total Recall) ≤ 35; (2) HVLT Recognition Discrimination Index ≤ 35; (3) Benton Judgment of Line Orientation ≤ 6; (4) Letter Number Sequencing ≤ 6; (5) Semantic Fluency Test ≤ 35; (6) Symbol Digit Modalities Test ≤ 35.

#### 2.3.4 Predictor selection and model development

A total of 16 candidate predictors covering demographic characteristics, disease-related features, motor symptoms, and non-motor manifestations were initially considered for model development based on clinical relevance and data availability. Detailed definitions, variable types, and coding schemes of all candidate predictors are provided in Supplementary Table S1.

#### 2.3.5 Feature selection

Feature selection was performed using complementary statistical and machine learning approaches. Univariate analysis was conducted solely to compare baseline characteristics between participants with and without EDS and was not used for variable preselection. Subsequently, all candidate predictors were independently entered into both least absolute shrinkage and selection operator (LASSO) regression and the Boruta algorithm. Predictors with non-zero coefficients identified by LASSO and variables confirmed by Boruta were compared, and the intersection of variables identified by both methods was retained as the final predictor set for model development.

##### 2.3.5.1 Univariate analysis

Prior to model development, univariate analyses were performed to compare baseline characteristics between participants with and without EDS. Categorical variables were compared using the chi-square test, whereas continuous variables were analyzed using independent t tests (23). Univariate analysis was performed for descriptive purposes only and was not used for variable preselection.

##### 2.3.5.2 LASSO regression and Boruta feature selection

Two complementary machine learning-based feature selection methods were independently applied to all candidate predictors, including LASSO regression and the Boruta algorithm. LASSO regression was performed with L1 regularization, and predictors with non-zero coefficients at λ_min were retained. Boruta feature selection was conducted using a random forest-based wrapper method, and only “confirmed” important variables were selected.

Finally, the intersection of variables identified by both methods was retained for subsequent model development.

#### 2.3.6 Model training

Four predictive algorithms were used to construct models for EDS, including logistic regression (LR), extreme gradient boosting (XGB), naive Bayes (NB), and light gradient boosting machine (LGBM). To ensure a fair comparison, all candidate models were developed and compared within an identical five-fold cross-validation framework. Hyperparameter tuning was performed within the cross-validation framework to improve model performance and reduce overfitting.

#### 2.3.7 Model interpretation

To enhance interpretability, we applied the SHAP framework, a game—theoretic approach that assigns each predictor a consistent, locally accurate contribution (SHAP value) to every individual prediction (24). These values quantify the direction and magnitude of each variable’s influence on the model output, thereby increasing transparency and facilitating comprehension of the decision process.

### 2.4 Statistical analysis

Data were analyzed using R software (version 4.4.2) and DCPM (V5.49, Jingding Medical Technology Co., Ltd.). For the analysis, only variables with less than 50% of missing values were considered. Remaining missing values were imputed using the median and the most frequent category, for numerical and categorical features, respectively. The PPMI dataset was randomly divided, with 70% allocated to the training dataset and 30% assigned to the internal validation dataset. Model development was performed using the training dataset, and performance was evaluated in the internal validation dataset. Categorical variables were presented as counts and percentages and compared using the chi-square test. The distribution of continuous variables was assessed for normality using the Kolmogorov–Smirnov test. Normally distributed continuous variables were expressed as mean ± standard deviation, and intergroup comparisons were performed using the *t* test. Non-normally distributed continuous variables were presented as median and interquartile range, with intergroup comparisons conducted using the rank-sum test. Baseline characteristics were compared using univariate analyses. Feature selection was subsequently performed using LASSO regression and the Boruta algorithm independently. Four predictive algorithms—LR, XGB, NB, and LGBM—were used for model training and validation. Calibration plots generated using the bootstrap method were used to assess agreement between predicted and observed probabilities, while decision curve analysis (DCA) was employed to evaluate the net clinical benefit of the model across a range of threshold probabilities (25, 26). A two-tailed *p* < 0.05 was considered statistically significant.

## 3 Results

### 3.1 Characteristics of the study population

This study enrolled 676 participants from PPMI, including 573 non-EDS cases and 103 EDS cases. A total of 180 individuals from the SPH-PD were ultimately included, including 122 non-EDS cases and 58 EDS cases. The baseline characteristics are presented in Supplementary Table S2.

### 3.2 Univariate analysis of predictive factors for EDS in the training dataset

Among the 473 participants in the training dataset, 69 had EDS. Seven variables showed statistically significant differences between patients with and without EDS in the univariate analysis, as shown in Supplementary Table S3. These predictors included MCI, GDS, STAI, SCOPA, UPDRS I, UPDRS II, and UPDRS III.

### 3.3 Construction and performance evaluation of risk prediction models for EDS

**Figure 2:**
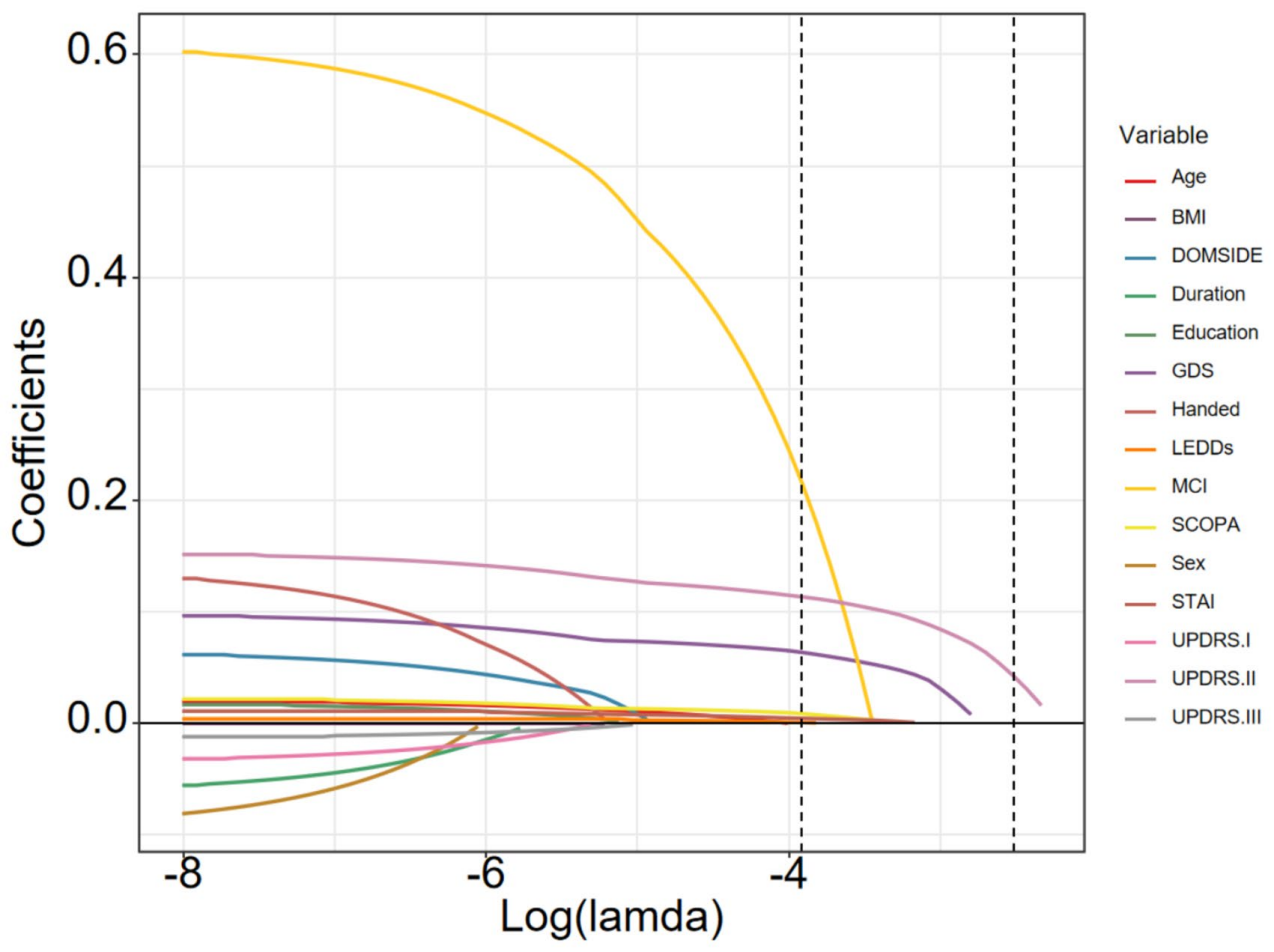
Select discriminative variables using the LASSO binary logistic regression model.

**Figure 3:**
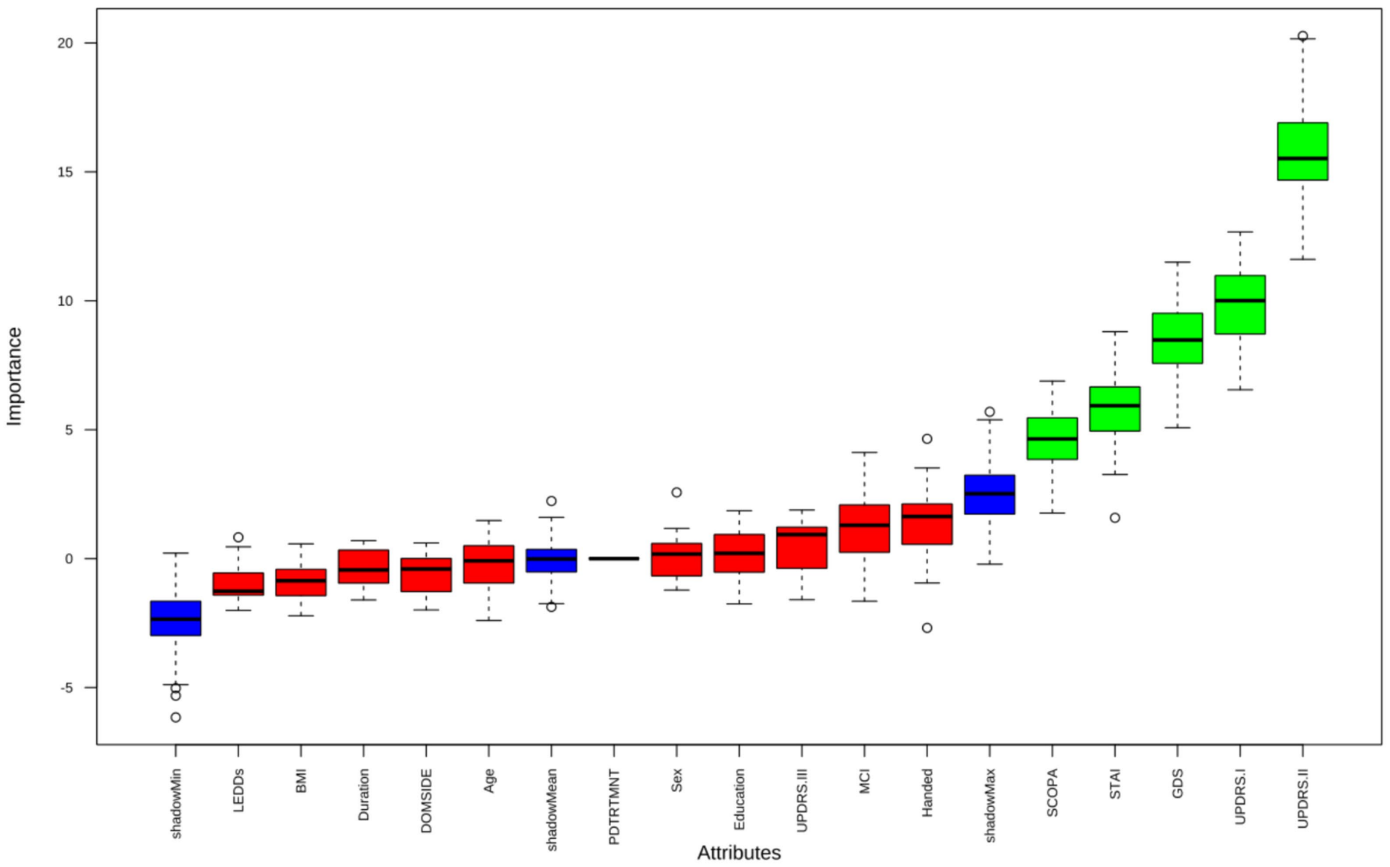
Feature selection using the Boruta algorithm. ShadowMin, shadowMean, and shadowMax represent artificial reference variables generated by Boruta and were not included in the final prediction model.

LASSO regression and the Boruta algorithm were independently applied to the same set of candidate predictors. Five variables consistently identified by both methods (GDS, STAI, SCOPA, UPDRS I, and UPDRS II) were selected as the final predictors for subsequent model development (Figures 2, 3). LASSO regression identified a subset of predictors with non-zero coefficients at λ_min, which were subsequently used for model development and validation. The Boruta algorithm was applied within an RF framework to further assess variable importance. Confirmed features were then incorporated into the machine learning models.

### 3.4 Model construction and discriminative power

**Figure 4:**
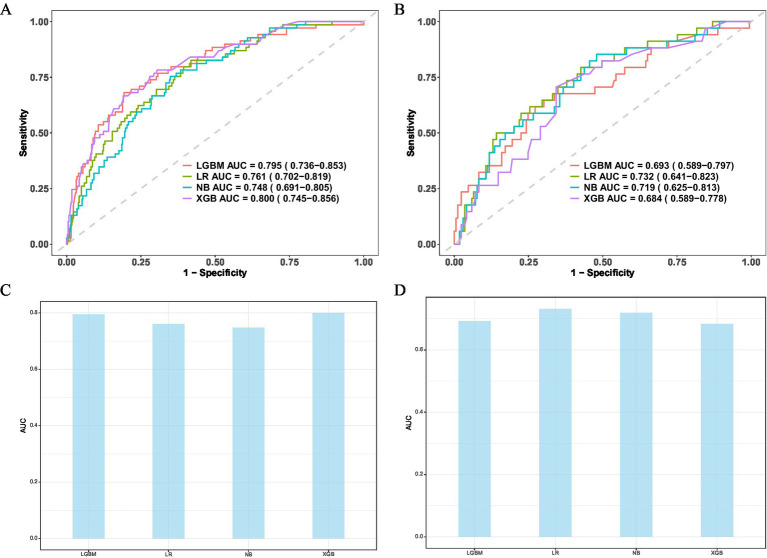
ROC curves and AUC of four ML models. **(A)** ROC curves of each model in the training dataset. **(B)** ROC curves of each model in the validation dataset. **(C)** AUC of each model in the training dataset. **(D)** AUC of each model in the validation dataset.

Four predictive models (LR, XGB, NB, and LGBM) were developed using the training dataset and evaluated in the validation dataset. Model performance was assessed using multiple metrics, including the area under the receiver operating characteristic curve, accuracy, specificity, recall, and brier. Detailed results are presented in Table 1, while the corresponding receiver operating characteristic curves are shown in Figure 4 and Supplementary Figure S1.

**Table 1:** Performance metrics of each model on the training dataset and validation dataset.

Model	Dataset	Accuracy	Recall	Specificity	Brier
LR	Training	0.6173	0.8261	0.5817	0.1108
XGB	Training	0.7082	0.7826	0.6955	0.1038
LGBM	Training	0.7886	0.6812	0.8069	0.1035
NB	Training	0.6660	0.7536	0.6510	0.1540
LR	Validation	0.6059	0.7647	0.5740	0.1321
XGB	Validation	0.6700	0.5294	0.6982	0.1351
LGBM	Validation	0.7389	0.4706	0.7929	0.1271
NB	Validation	0.6207	0.7059	0.6036	0.1646

LR, logistic regression; XGB, extreme gradient boosting; NB, naive Bayes; LGBM, gradient boosting machine.

### 3.5 Cross-validation and model stability

**Figure 5:**
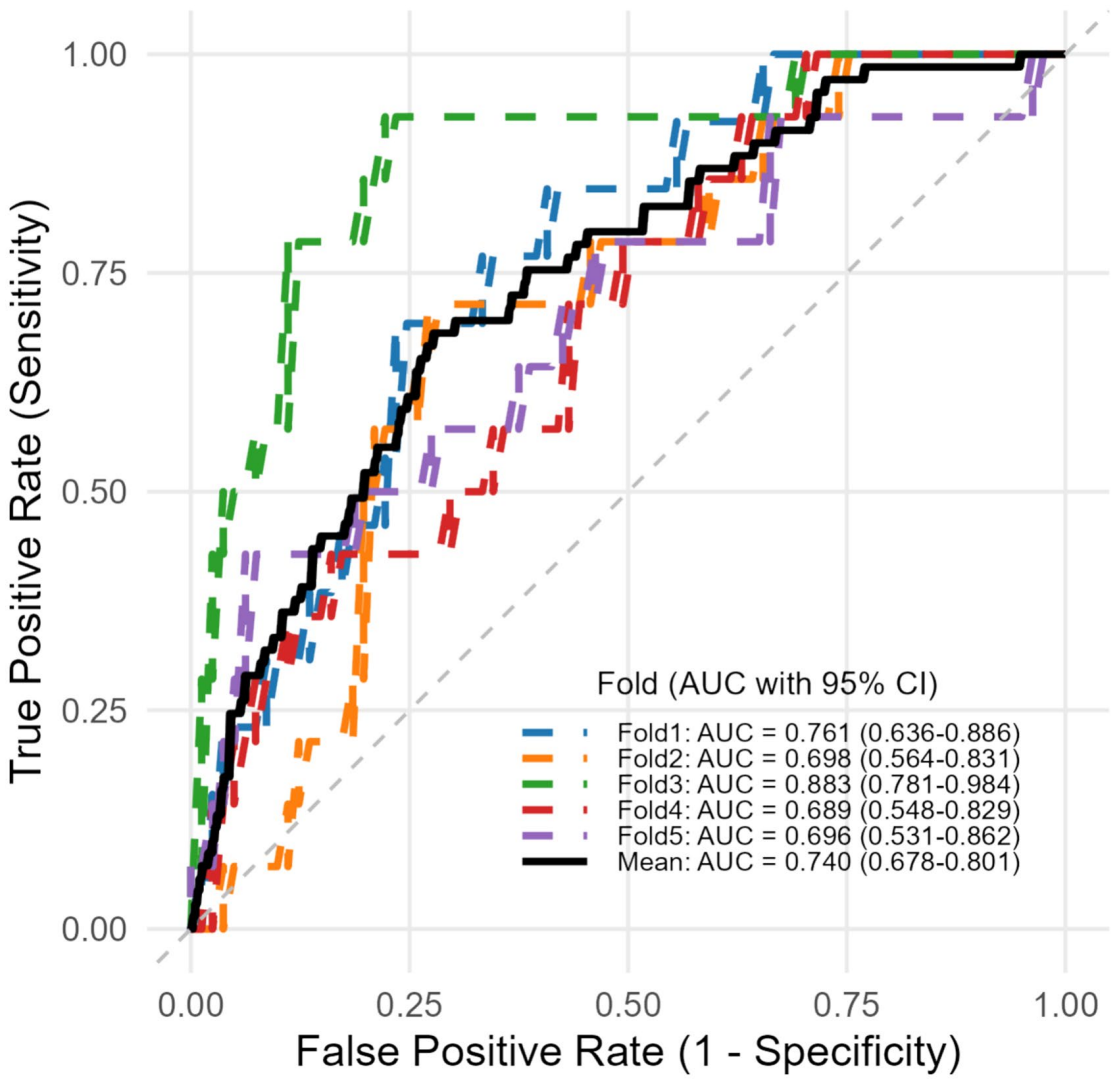
Five-fold cross-validation of the training dataset.

Model performance differed across the validation dataset. In the internal validation dataset, the LR model achieved the highest AUC (0.732), indicating superior discriminative performance compared with the other candidate models. Within the unified five-fold cross-validation framework, no consistently dominant advantage of the more complex algorithms over LR was observed (Figure 5).

Regarding calibration, LGBM yielded the lowest brier score, although the difference compared with LR was marginal. Pairwise comparisons of ROC AUCs using DeLong’s test showed no statistically significant differences among the models, suggesting comparable discriminatory ability. Therefore, considering discrimination in the validation dataset, cross-validation stability, generalization ability, calibration performance, and model interpretability, the LR model was ultimately selected as the optimal predictive model.

### 3.6 Clinical utility assessment

The clinical utility of each model was evaluated using DCA and precision–recall (PR) curves. In the training dataset, all models demonstrated positive net benefit over the “treat-all” and “treat-none” strategies across a wide range of threshold probabilities. Among them, the XGB and LGBM models showed slightly higher net benefit at moderate thresholds, whereas the NB model performed relatively poorly (Supplementary Figure S2).

In the validation dataset, the overall net benefit of all models decreased compared with the training dataset. Notably, the LR model demonstrated more stable and consistent net benefit across a broader range of clinically relevant threshold probabilities, while the performance of XGB declined substantially, indicating limited generalizability. LGBM showed moderate performance, whereas NB remained inferior.

**Figure 6:**
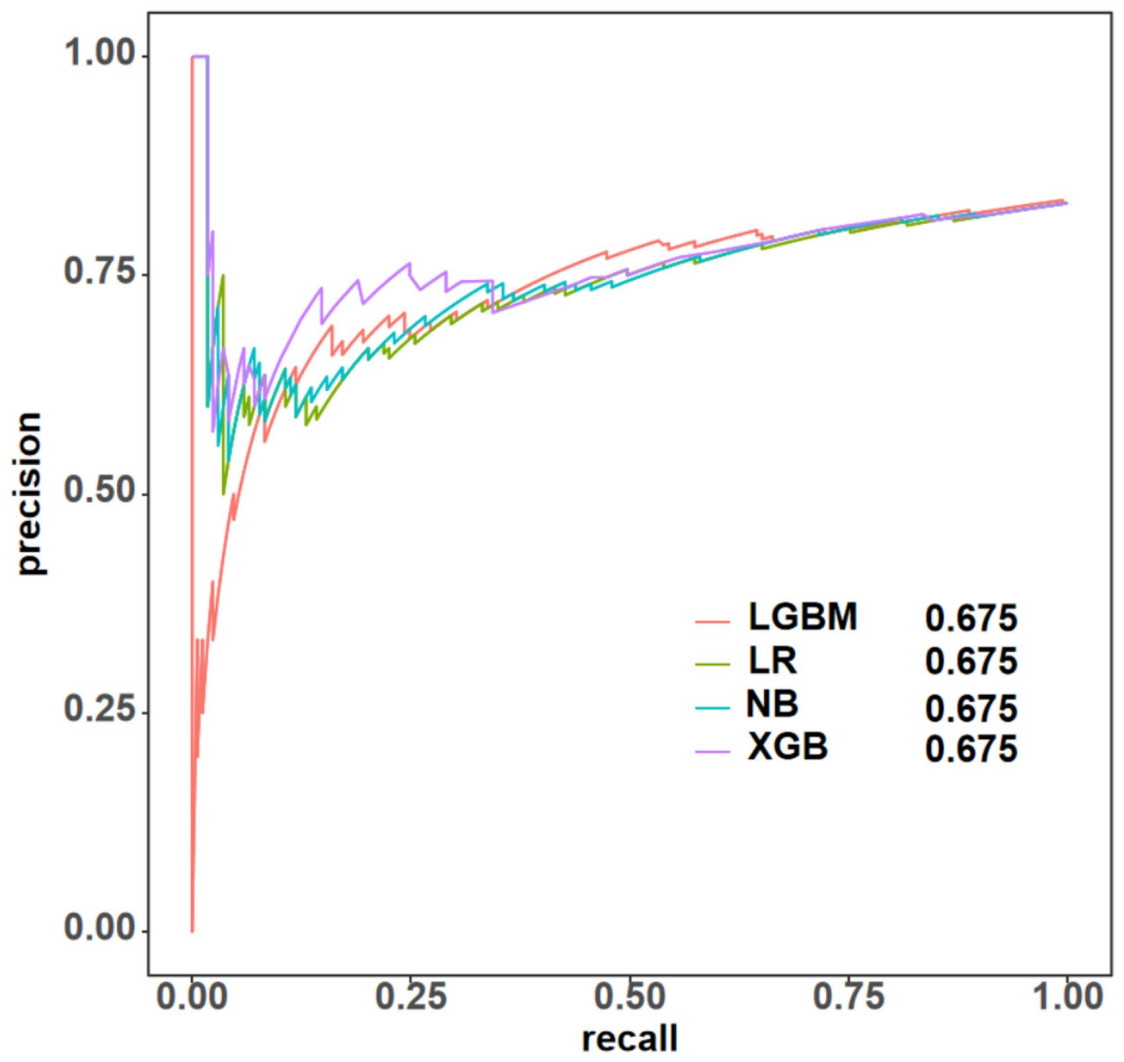
The PR curve and PRAUC of the validation dataset.

Consistent with the DCA results, PR curve analysis demonstrated comparable precision–recall trade-offs among the models, with no substantial advantage observed for complex machine learning models over LR in the validation dataset (Figure 6). Therefore, although boosting-based models exhibited slightly higher net benefit in the training dataset, the LR model showed superior stability and generalizability in the validation dataset, suggesting better generalizability and overall model stability. These findings suggest that the LR model may support clinical decision-making and demonstrate potential applicability in real-world settings.

### 3.7 External validation

The performance of the final model was further evaluated in an external validation cohort. The ROC analysis showed that the model achieved an AUC of 0.673 (95% CI: 0.552–0.793), indicating moderate discriminative ability in an independent dataset (Supplementary Figure S3).

To further evaluate model performance in the context of class imbalance, additional classification metrics were calculated, including accuracy, balanced accuracy, F1-score, and precision-recall area under the curve (PR-AUC). These results are summarized in Supplementary Figure S4; Supplementary Table S4. Overall, the model demonstrated balanced classification performance across multiple evaluation metrics.

DCA demonstrated that the model provided a positive net benefit across a range of clinically relevant threshold probabilities, outperforming both the “treat-all” and “treat-none” strategies within this interval. However, the net benefit decreased at higher threshold probabilities, suggesting that the clinical utility of the model may be limited in more stringent decision-making scenarios (Supplementary Figure S3).

Overall, these findings indicate that the model maintained moderate discriminative ability in an independent cohort, although additional validation and refinement are required before broader clinical application.

### 3.8 Model interpretation and individualized prediction

**Figure 7:**
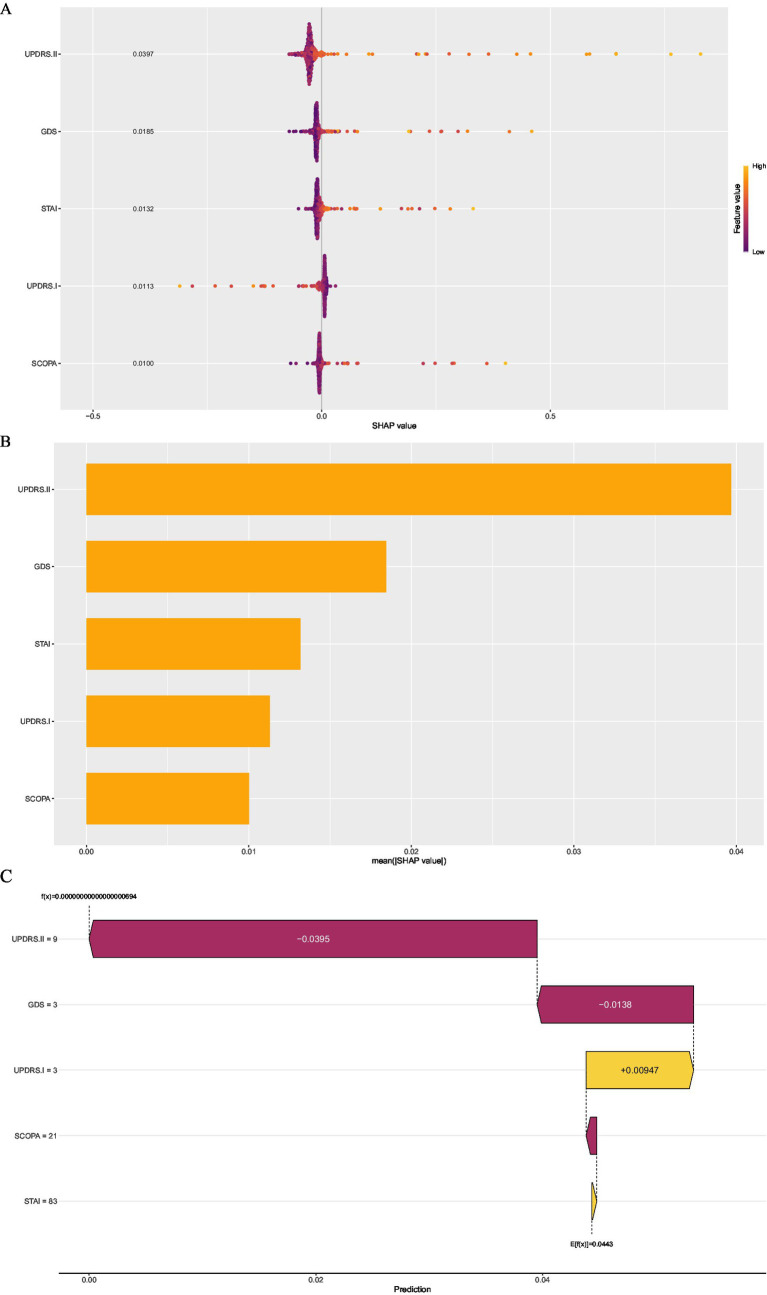
Feature interpretation in LR models. **(A)** Global feature contribution analysis showing the magnitude and direction of each predictor’s influence on model output. **(B)** Ranking of feature importance in the LR model. **(C)** Individual SHAP waterfall plot for a representative patient. The selected patient had UPDRS II score of 9, GDS score of 3, UPDRS I score of 3, SCOPA-AUT score of 21, and STAI score of 83. The baseline model prediction was 0.0443, and the final individualized prediction was close to 0. Positive SHAP values increased the predicted probability of EDS, whereas negative SHAP values decreased the predicted probability.

To enhance model interpretability, SHAP analysis was performed to quantify both the global and individual contributions of each predictor. The SHAP summary bar plot demonstrated that UPDRS II was the most influential variable in the model, with the highest mean absolute SHAP value (0.0397), indicating its largest contribution among included predictors to prediction. This was followed by GDS (0.0185) and STAI (0.0132), while UPDRS I (0.0113) and SCOPA (0.0100) showed relatively smaller but still meaningful contributions (Figures 7A,B). Overall, motor symptom burden and neuropsychiatric features jointly contributed to the model output, with UPDRS II playing an important role.

The SHAP beeswarm plot further illustrated the distribution and directionality of feature effects across individuals. Higher values of UPDRS II, GDS, and STAI were generally associated with positive SHAP values, indicating an increased predicted risk (Figure 7C). In contrast, lower feature values tended to shift predictions toward lower risk (negative SHAP values).

Notably, UPDRS II exhibited the widest spread of SHAP values, suggesting substantial heterogeneity in its impact across patients and reinforcing its role as the most influential predictor in this model. Other variables showed narrower distributions, indicating more modest but consistent effects.

To further illustrate individual-level interpretability, SHAP waterfall analysis was performed for a representative patient (UPDRS II = 9, GDS = 3, UPDRS I = 3, SCOPA-AUT = 21, STAI = 83). Lower values of UPDRS II and GDS contributed negatively to the predicted probability, resulting in a low estimated risk of EDS

## 4 Discussion

PD represents a progressive neurodegenerative disorder imposing substantial global burdens through motor disability and complex non-motor manifestations. Among these, EDS emerges as a prevalent yet frequently under-recognized symptom that severely compromises patient quality of life, daily functioning, and safety (27, 28). Despite its clinical significance, EDS is often insufficiently characterized within the broader clinical spectrum of PD. Identifying multidimensional clinical factors associated with EDS may facilitate earlier recognition of patients who require closer monitoring and comprehensive sleep evaluation.

In this study, we developed and externally validated an explainable machine learning model for EDS risk stratification in patients with H&Y stage 1 PD. By integrating statistical assessment, comparison of multiple predictive algorithms, independent external validation, and SHAP-based interpretability analysis, we identified several important findings. The main findings were as follows: first, EDS in H&Y stage 1 PD was associated with multidimensional clinical features rather than a single domain; second, systematic comparison of multiple predictive algorithms showed that a relatively simple and interpretable LR model achieved the best balance between predictive performance and interpretability; and third, explainability analysis confirmed that the most influential predictors were clinically plausible and aligned with current understanding of non-motor symptom burden in PD.

The observed prevalence of EDS in our early-stage PD cohort, while lower than 50% range often cited in heterogeneous populations, aligns with findings suggesting that subjective sleepiness may not always correlate with objective nocturnal sleep disturbances measured by polysomnography (29). This discrepancy likely stems from the specific pathophysiology of EDS in PD, which recent neuroimaging studies indicate is driven more by dysfunction in wake-promoting pathways and the default mode network rather than solely by fragmented nocturnal sleep architecture (30, 31). Specifically, altered amplitude of low-frequency fluctuations in the pons and posterior cingulate cortex has been linked to EDS severity, supporting the hypothesis that central arousal mechanisms are compromised independently of peripheral sleep quality (30, 31). Furthermore, the association between EDS and parkinsonian-like symptoms even in middle-aged adults suggests that sleepiness may be an early marker of neurodegeneration preceding overt motor diagnosis, potentially reflecting early brainstem involvement (32).

Many prediction studies achieve promising performance in internal validation datasets but fail to demonstrate robustness in independent populations. External validation is therefore a critical step in evaluating model transportability and potential clinical relevance. In the present study, the selected model achieved moderate discrimination in the independent external validation cohort. Although the performance was lower than that observed in the development dataset, external validation across different populations provides a more realistic assessment of model transportability. Differences in education level, demographic characteristics, and clinical settings between the PPMI and external cohort may have contributed to reduced predictive performance. Therefore, the current model should be considered as a preliminary risk stratification framework rather than a definitive diagnostic tool.

In addition, interpretability is another key issue in clinical machine learning. A model with limited transparency may be difficult to implement in clinical practice. To address this concern, we used SHAP analysis to quantify feature contributions and visualize how specific variables influenced model output. The results indicated that GDS, STAI, SCOPA, UPDRS I, and UPDRS II were the most influential predictors. These observations are consistent with the current understanding of the multidimensional pathophysiology of EDS in PD. Unlike ESS, which directly evaluates the current subjective tendency to fall asleep and was used as the outcome definition in this study, the predictors included in our model capture broader PD-related clinical domains rather than sleepiness itself. Specifically, GDS and STAI represent mood-related burden, SCOPA reflects autonomic dysfunction, and UPDRS I and II characterize non-motor experiences and motor-related functional impairment in daily living. These multidimensional features may provide complementary information regarding EDS-related clinical phenotypes beyond the assessment of daytime sleepiness severity alone. Together, these findings support the biological plausibility and clinical relevance of the identified predictors.

Our study also has practical implications. Because the model is based on routinely obtainable clinical variables, it may be feasible for use in neurology clinics and movement disorder centers without requiring advanced imaging or specialized laboratory biomarkers. The model is not intended to replace ESS-based assessment but may serve as a complementary tool to characterize EDS-related clinical profiles and support comprehensive evaluation of sleep-related problems in early-stage PD. Clinicians may use the model as an auxiliary tool to identify patients who may benefit from more detailed sleep assessment, medication review, behavioral intervention, or multidisciplinary management. In this sense, the model may serve as a supportive framework to complement existing clinical assessments and facilitate individualized management of sleep-related problems in patients with H&Y stage 1 PD.

However, several limitations warrant consideration. First, the reliance on the ESS as the sole measure of EDS introduces potential subjective bias, as ESS scores may be influenced by recall inaccuracies or comorbid mood disturbances; future studies incorporating objective polysomnography (PSG) or the Multiple Sleep Latency Test (MSLT) could enhance diagnostic precision. Second, the study’s external validation cohort, although independent, was limited in size (*n* = 180) and derived from a predominantly early-stage PD population (H&Y stage 1), which may limit model transportability to advanced or more diverse ethnic populations. Third, the absence of neuroimaging biomarkers (e.g., dopamine transporter SPECT or cerebrospinal fluid *α*-synuclein) may have precluded capturing underlying pathological substrates critical for a fully mechanistic interpretation of EDS risk. Finally, although the prediction framework demonstrated potential value, the moderate external validation performance indicates that further optimization and multicenter validation are required.

Future research should focus on prospective multicenter validation, refinement of sleep phenotype classification, and longitudinal validation of the proposed risk stratification framework. Specifically, future longitudinal studies are needed to determine whether baseline neuropsychiatric, autonomic, and motor profiles can predict the development of incident EDS during disease progression. It would also be valuable to explore whether integration of wearable monitoring, neuroimaging markers, or biological indicators can further enhance predictive accuracy while maintaining clinical usability.

## 5 Conclusion

We developed and externally validated an explainable prediction model for EDS risk stratification in patients with H&Y stage 1 PD. By integrating multidimensional clinical information and SHAP-based interpretation, the model demonstrated moderate discriminative performance and good interpretability. Although further prospective validation in larger and more diverse populations is required, this framework may support preliminary risk stratification and comprehensive clinical assessment of sleep-related problems in early-stage PD.

## Data Availability

The PPMI data analyzed in this study are publicly available from the PPMI database (https://www.ppmi-info.org/access-data-specimens/download-data) upon registration and approval by PPMI. The external validation data generated during this study are available from the corresponding author upon reasonable request.

## Glossary

PPMI: Parkinson Progression Markers Initiative
EDS: Excessive daytime sleepiness
H&Y: Hoehn and Yahr
SHAP: The Shapley Additive Explanations
SPECT: Single-photon emission computed tomography
UPDRS: Unified Parkinson’s Disease Rating Scale
ESS: Epworth Sleepiness Scale
BMI: Body mass index
PD: Parkinson’s disease
DOMSIDE: Dominant side of motor symptoms
PDTRTMNT: PD treatment status
MCI: Mild cognitive impairment
GDS: Geriatric Depression Scale
STAI: State–Trait Anxiety Index
SCOPA: Scales for Outcomes in Parkinson’s Disease-Autonomic
RH: Right-handed
LH: Left-handed
MH: Mixed-handed
SD: Standard deviation
HVLT-Total Recall: Hopkins verbal learning test-total recall
LASSO: Least Absolute Shrinkage and Selection Operator
RF: Random forest
LR: Logistic regression
XGB: Extreme gradient boosting
NB: Naive Bayes
LGBM: Light gradient boosting machine
PSG: Polysomnography
MSLT: Multiple Sleep Latency Test
AUROC: Area under the receiver operating characteristic curve
DCA: Decision curve analysis;

## References

[1] Liu H Li J Wang X Huang J Wang T Lin Z . Excessive daytime sleepiness in Parkinson's disease. Nat Sci Sleep. (2022) 14:1589–609. doi: 10.2147/NSS.S375098, 36105924 PMC9464627

[2] Kobak Tur E Demir M Kenangil G Mayda Domaç F. Sleep quality, excessive daytime sleepiness, and depression in Parkinson's disease: implications for improved patient outcomes. Neurol Res. (2024) 46:297–303. doi: 10.1080/01616412.2024.2301878, 38264903

[3] Du S. Qin Y. Han M. Huang Y. Cui J. Han H. Longitudinal mediating effect of depression on the relationship between excessive daytime sleepiness and activities of daily living in Parkinson's disease, Clin Gerontol (2024) 47:426–435. doi: 10.1080/07317115.2022.2111014 35951004

[4] Huang Y. Du S. Chen D. Qin Y. Cui J. Han H. The path linking excessive daytime sleepiness and activity of daily living in Parkinson's disease: the longitudinal mediation effect of autonomic dysfunction, Neurol Sci (2022) 43:4777–4784. doi: 10.1007/s10072-022-06081-0 35487997

[5] Sportelli C Poplawska-Domaszewicz K Borley C Metta V Leta V Wu K . "dozing off" in the car and excessive daytime sleepiness (EDS) in Parkinson's disease: a survey of 125 patients. J Neural Transm (Vienna). (2026) 133:589–93. doi: 10.1007/s00702-025-02995-z, 40931261

[6] Feng F Cai Y Hou Y Ou R Jiang Z Shang H. Excessive daytime sleepiness in Parkinson's disease: a systematic review and meta-analysis. Parkinsonism Relat Disord. (2021) 85:133–40. doi: 10.1016/j.parkreldis.2021.02.016, 33637423

[7] Del Pino R. Murueta-Goyena A. Ayala U. Acera M. Fernández M. Tijero B. Clinical long-term nocturnal sleeping disturbances and excessive daytime sleepiness in Parkinson's disease, PLoS One (2021) 16:e0259935. doi: 10.1371/journal.pone.0259935 34851977 PMC8635374

[8] Mahajan A Duque KR Dwivedi AK Abanto J Marsili L Hill EJ . Exploring the intersection between orthostatic hypotension and daytime sleepiness in Parkinson's disease. J Neurol Sci. (2025) 468:123366. doi: 10.1016/j.jns.2024.123366, 39740578

[9] Iijima M Osawa M Yasuda S Kitagawa K. Association between excessive daytime sleepiness and the cholinergic ascending reticular system in Parkinson's disease. Neurodegener Dis. (2021) 21:48–54. doi: 10.1159/000519776, 34564079

[10] Hu Y Guo P Lian TH Zuo LJ Yu SY Liu L . Clinical characteristics, Iron metabolism and Neuroinflammation: new insight into excessive daytime sleepiness in Parkinson's disease. Neuropsychiatr Dis Treat. (2021) 17:2041–51. doi: 10.2147/NDT.S272110, 34188474 PMC8232841

[11] Lin J Li C Ou R Hou Y Zhang L Wei Q . Longitudinal evolution and plasma biomarkers for excessive daytime sleepiness in Parkinson's disease. J Gerontol A Biol Sci Med Sci. (2024) 79:glae086 [pii]. doi: 10.1093/gerona/glae086, 38526870

[12] Chitsaz A Najafi MR Habibi F Amirhajloo S. Comparison of the effectiveness of modafinil and methylphenidate in treatment of excessive daytime sleepiness in patients with Parkinson's disease. Curr J Neurol. (2024) 23:39–43. doi: 10.18502/cjn.v23i1.16431, 39431232 PMC11489626

[13] Corvol JC Azulay JP Bosse B Dauvilliers Y Defebvre L Klostermann F . THN 102 for excessive daytime sleepiness associated with Parkinson's disease: a phase 2a trial. Mov Disord. (2022) 37:410–5. doi: 10.1002/mds.28840, 34709684

[14] Videnovic A Amara AW Comella C Schweitzer PK Emsellem H Liu K . Solriamfetol for excessive daytime sleepiness in Parkinson's disease: phase 2 proof-of-concept trial. Mov Disord. (2021) 36:2408–12. doi: 10.1002/mds.28702, 34191352 PMC8596433

[15] Deng P Xu K Zhou X Xiang Y Xu Q Sun Q . Constructing prediction models for excessive daytime sleepiness by nomogram and machine learning: a large Chinese multicenter cohort study. Front Aging Neurosci. (2022) 14:938071. doi: 10.3389/fnagi.2022.938071, 35966776 PMC9372350

[16] Liu Y Xue L Zhao J Dou K Wang G Xie A. Clinical characteristics in early Parkinson's disease with excessive daytime sleepiness: a cross-sectional and longitudinal study. Clin Transl Sci. (2023) 16:2033–45. doi: 10.1111/cts.13610, 37551840 PMC10582661

[17] Hassan A Ahmed A. Predicting Parkinson's disease progression: a non-invasive method leveraging voice inputs. JCS. (2023) 8:66–82. doi: 10.53070/bbd.1350356

[18] Parkinson Progression Marker Initiative. The Parkinson progression marker initiative (PPMI). Prog Neurobiol. (2011) 95:629–35. doi: 10.1016/j.pneurobio.2011.09.005, 21930184 PMC9014725

[19] Hughes AJ Daniel SE Kilford L Lees AJ. Accuracy of clinical diagnosis of idiopathic Parkinson's disease: a clinico-pathological study of 100 cases. J Neurol Neurosurg Psychiatry. (1992) 55:181–4. doi: 10.1136/jnnp.55.3.181, 1564476 PMC1014720

[20] Goetz CG Tilley BC Shaftman SR Stebbins GT Fahn S Martinez-Martin P . Movement Disorder Society-sponsored revision of the unified Parkinson's disease rating scale (MDS-UPDRS): scale presentation and clinimetric testing results. Mov Disord. (2008) 23:2129–70. doi: 10.1002/mds.22340, 19025984

[21] Johns MW. A new method for measuring daytime sleepiness: the Epworth sleepiness scale. Sleep. (1991) 14:540–5. doi: 10.1093/sleep/14.6.540, 1798888

[22] Johns MW. Sensitivity and specificity of the multiple sleep latency test (MSLT), the maintenance of wakefulness test and the Epworth sleepiness scale: failure of the MSLT as a gold standard. J Sleep Res. (2000) 9:5–11. doi: 10.1046/j.1365-2869.2000.00177.x, 10733683

[23] Fan L Yu Z Liu Y Zhu H Wang L. Development and validation of a risk prediction nomogram for frailty in older Chinese adults. J Health Popul Nutr. (2025) 44:404. doi: 10.1186/s41043-025-01148-y, 41267110 PMC12632084

[24] Thimmapuram M Akepogu AR Radhika Raju P. Analysis of freezing of gait in Parkinson's disease detection using a multimodal prototype learning framework. IEEE Trans Neural Syst Rehabil Eng. (2025) 33:3709–22. doi: 10.1109/TNSRE.2025.3605204, 40892658

[25] Alba AC Agoritsas T Walsh M Hanna S Iorio A Devereaux PJ . Discrimination and calibration of clinical prediction models: users' guides to the medical literature. JAMA. (2017) 318:1377–84. doi: 10.1001/jama.2017.12126 29049590

[26] Vickers AJ Cronin AM Elkin EB Gonen M. Extensions to decision curve analysis, a novel method for evaluating diagnostic tests, prediction models and molecular markers. BMC Med Inform Decis Mak. (2008) 8:53. doi: 10.1186/1472-6947-8-53, 19036144 PMC2611975

[27] Maggi G Vitale C Cerciello F Santangelo G. Sleep and wakefulness disturbances in Parkinson's disease: a meta-analysis on prevalence and clinical aspects of REM sleep behavior disorder, excessive daytime sleepiness and insomnia. Sleep Med Rev. (2023) 68:101759. doi: 10.1016/j.smrv.2023.101759, 36708642

[28] Murasan I Diaconu S Bougea A Falup-Pecurariu C. Excessive daytime sleepiness in Parkinson's disease and parkinsonism. Sleep Med Clin. (2025) 20:333–41. doi: 10.1016/j.jsmc.2025.06.002, 40912747

[29] Dodet P Houot M Leu-Semenescu S Corvol JC Lehéricy S Mangone G . Sleep disorders in Parkinson's disease, an early and multiple problem. NPJ Parkinsons Dis. (2024) 10:46. doi: 10.1038/s41531-024-00642-0, 38424131 PMC10904863

[30] Rosinvil T Postuma RB Rahayel S Bellavance A Daneault V Montplaisir J . Clinical symptoms and neuroanatomical substrates of daytime sleepiness in Parkinson's disease. NPJ Parkinsons Dis. (2024) 10:149. doi: 10.1038/s41531-024-00734-x, 39122721 PMC11316005

[31] Wang X Wang M Yuan Y Li J Shen Y Zhang K. Altered amplitude of low-frequency fluctuations and functional connectivity in excessive daytime sleepiness in Parkinson disease. Front Neurosci. (2020) 14:29. doi: 10.3389/fnins.2020.00029, 32082108 PMC7006219

[32] Shin C Kim R Thomas RJ Yun CH Lee SK Abbott RD. Severity of daytime sleepiness and parkinsonian-like symptoms in Korean adults aged 50-64 years. J Clin Neurol. (2022) 18:33–40. doi: 10.3988/jcn.2022.18.1.33, 35021274 PMC8762500

