# Supplementary material for: Machine learning–based prediction of excessive daytime sleepiness in patients with Parkinson’s disease: findings from the PPMI cohort with external validation
Source: Front Med (Lausanne). 2026 Jul 20;13:1894008. doi: 10.3389/fmed.2026.1894008 (PMC13431447; doi:10.3389/fmed.2026.1894008)
Supplement: Supplementary file 1 [file Data_Sheet_1.zip › Revised version of Supplementary materials/Supplementary Figure S2.doc]

A	B

**Supplementary Figure S2**. DCA curves for four ML models. A. DCA curve in the training dataset. B. DCA curve in the validation dataset.
