# Supplementary material for: Machine learning–based prediction of excessive daytime sleepiness in patients with Parkinson’s disease: findings from the PPMI cohort with external validation
Source: Front Med (Lausanne). 2026 Jul 20;13:1894008. doi: 10.3389/fmed.2026.1894008 (PMC13431447; doi:10.3389/fmed.2026.1894008)
Supplement: Supplementary file 1 [file Data_Sheet_1.zip › Revised version of Supplementary materials/Supplementary Figure S4.doc]

**Supplementary Figure S4. Heatmap comparing classification performance across different predictive models in the external validation cohort.** Performance was evaluated using sensitivity, specificity, precision, recall, F1-score, positive predictive value (PPV), and negative predictive value (NPV). The heatmap provides a comprehensive comparison of classification performance beyond ROC-AUC.
