# Supplementary material for: Machine learning–based prediction of excessive daytime sleepiness in patients with Parkinson’s disease: findings from the PPMI cohort with external validation
Source: Front Med (Lausanne). 2026 Jul 20;13:1894008. doi: 10.3389/fmed.2026.1894008 (PMC13431447; doi:10.3389/fmed.2026.1894008)
Supplement: Supplementary file 1 [file Data_Sheet_1.zip › Revised version of Supplementary materials/Supplementary Table S1.doc]

**Supplementary Table S1**. Definitions and coding schemes of candidate predictors used for model development.

| **Feature** | **Definition** | **Type** | **Coding/Unit** |
| --- | --- | --- | --- |
| Age | Age at baseline assessment | Continuous | Years |
| Sex | Biological sex | Categorical | Male/Female |
| Education | Years of formal education | Continuous | Years |
| Handed | Dominant hand preference | Categorical | Right-handed / Left-handed / Mixed-handed |
| BMI | Body mass index at baseline assessment | Continuous | kg/m² |
| Disease duration | Time since PD diagnosis | Continuous | Years |
| DOMSIDE | Side with predominant parkinsonian motor manifestations | Categorical | Left / Right / Symmetric |
| PDTRTMNT | Use of anti-parkinsonian medication at baseline | Categorical | No / Yes |
| LEDDs | Levodopa equivalent daily dose | Continuous | mg/day |
| MCI | Mild cognitive impairment status | Categorical | No / Yes |
| GDS | Geriatric Depression Scale score | Continuous | Total score |
| STAI | State-Trait Anxiety Inventory score | Continuous | Total score |
| SCOPA | Scale for Outcomes in Parkinson's Disease-Autonomic score | Continuous | Total score |
| UPDRS I | Non-motor experiences of daily living | Continuous | Total score |
| UPDRS II | Motor experiences of daily living | Continuous | Total score |
| UPDRS III | Motor examination score | Continuous | Total score |

BMI: Body Mass Index; DOMSIDE: dominant side of motor symptoms; PDTRTMNT: PD treatment status; LEDDs: levodopa doses; MCI: mild cognitive impairment; GDS: Geriatric Depression Scale; STAI: State-Trait Anxiety Index; SCOPA: Scales for Outcomes in Parkinson’s Disease-Autonomic; UPDRS: Unified Parkinson’s Disease Rating Scale.
