# Supplementary material for: Machine learning–based prediction of excessive daytime sleepiness in patients with Parkinson’s disease: findings from the PPMI cohort with external validation
Source: Front Med (Lausanne). 2026 Jul 20;13:1894008. doi: 10.3389/fmed.2026.1894008 (PMC13431447; doi:10.3389/fmed.2026.1894008)
Supplement: Supplementary file 1 [file Data_Sheet_1.zip › Revised version of Supplementary materials/Supplementary Table S2.doc]

**Supplementary Table S2**. Baseline characteristics of patients with PD

| **Variable** | **PPMI database** | | | | | | | | **SPH-PD database** | | | |
| --- | --- | --- | --- | --- | --- | --- | --- | --- | --- | --- | --- | --- |
|  | **Training dataset** | | | | **Internal validation dataset** | | | | **External validation dataset** | | | |
|  | ALL  N=473 | Non-EDS N=404 | EDS  N=69 | ***P*** | ALL N=203 | Non-EDS N=169 | EDS  N=34 | ***P*** | ALL N=180 | Non-EDS N=122 | EDS  N=58 | ***P*** |
| **Age_yrs** | 65.27 [59.31;71.14] | 65.06 [59.22;71.11] | 66.54 [60.59;72.29] | **0.269** | 66.69 [60.19;70.69] | 66.49 [59.88;70.36] | 68.35 [61.09;71.52] | **0.479** | 66  [60;71] | 66  [58;68] | 67  [65;75] | **0.003** |
| **Sex:** |  |  |  | **0.935** |  |  |  | **0.077** |  |  |  | **1.000** |
| Female | 170  (35.94%) | 146  (36.14%) | 24  (34.78%) |  | 65  (32.02%) | 59  (34.91%) | 6  (17.65%) |  | 85  (47.22%) | 58  (47.54%) | 27  (46.55%) |  |
| Male | 303  (64.06%) | 258  (63.86%) | 45  (65.22%) |  | 138  (67.98%) | 110  (65.09%) | 28  (82.35%) |  | 95  (52.78%) | 64  (52.46%) | 31  (53.45%) |  |
| **Education_yrs** | 16  [14;18] | 16  [14;18] | 16  [14;18] | **0.748** | 16  [15;18] | 16  [15;18] | 16.5  [16;18] | **0.984** | 9  [6;12] | 9  [6;12] | 9  [8;12] | **0.184** |
| **Handed:** |  |  |  | **0.538** |  |  |  | **0.261** |  |  |  | **1.000** |
| RH | 412  (87.10%) | 352  (87.13%) | 60  (86.96%) |  | 185  (91.13%) | 156  (92.31%) | 29  (85.29%) |  | 165  (91.67%) | 113  (92.62%) | 52  (89.66%) |  |
| LH | 49  (10.36%) | 43  (10.64%) | 6  (8.70%) |  | 9  (4.43%) | 7  (4.14%) | 2  (5.88%) |  | 10  (5.56%) | 6  (4.92%) | 4  (6.90%) |  |
| MH | 12  (2.54%) | 9  (2.23%) | 3  (4.35%) |  | 9  (4.43%) | 6  (3.55%) | 3  (8.82%) |  | 5  (2.78%) | 3  (2.46%) | 2  (3.45%) |  |
| **BMI** | 26.23 [23.97;29.00] | 26.23 [23.87;28.99] | 26.50 [24.24;29.06] | **0.538** | 26.02 [23.79;29.44] | 25.96 [23.67;29.58] | 26.37 [24.03;28.41] | **0.991** | 23.50 [21.05;25.95] | 23.19 [21.17;25.48] | 23.56 [20.78;27.34] | **0.385** |
| **Duration_yrs** | 0.54 [0.30;1.06] | 0.54 [0.32;1.06] | 0.54 [0.25;1.07] | **0.749** | 0.55 [0.34;1.06] | 0.55 [0.33;1.09] | 0.58 [0.39;0.95] | **0.891** | 0.55 [0.43;0.74] | 0.56 [0.44;0.73] | 0.54 [0.42;0.81] | **0.683** |
| **DOMSIDE:** |  |  |  | **0.219** |  |  |  | **0.028** |  |  |  | **0.229** |
| Left | 221  (46.72%) | 191  (47.28%) | 30  (43.48%) |  | 77  (37.93%) | 65  (38.46%) | 12  (35.29%) |  | 72  (40.00%) | 52  (42.62%) | 20  (34.48%) |  |
| Right | 239  (50.53%) | 204  (50.50%) | 35  (50.72%) |  | 122 (60.10%) | 103  (60.95%) | 19  (55.88%) |  | 83  (46.11%) | 51  (41.80%) | 32  (55.17%) |  |
| Symmetric | 13  (2.75%) | 9  (2.23%) | 4  (5.80%) |  | 4  (1.97%) | 1  (0.59%) | 3  (8.82%) |  | 25  (13.89%) | 19  (15.57%) | 6  (10.34%) |  |
| **PDTRTMNT:** |  |  |  | **- .** |  |  |  | **- .** |  |  |  | **0.535** |
| No | 473 (100.00%) | 404 (100.00%) | 69  (100.00%) |  | 203 (100.00%) | 169 (100.00%) | 34  (100.00%) |  | 21  (11.67%) | 19  (15.57%) | 2  (3.45%) |  |
| Yes | - | - | - |  | - | - | - |  | 159  (88.33%) | 103  (84.43%) | 56  (96.55%) |  |
| **LEDDs** | - | - | - | **-** | - | - | - |  | 473.52 [300.0;637.5] | 473.03 [300.0;625.0] | 507.50 [400.0;689.6] | **0.063-** |
| **MCI:** |  |  |  | **0.025** |  |  |  | **0.857** |  |  |  | **0.044** |
| NC | 402  (84.99%) | 350  (86.63%) | 52  (75.36%) |  | 168  (82.76%) | 139  (82.25%) | 29  (85.29%) |  | 57  (31.67%) | 45  (36.89%) | 12  (20.69%) |  |
| TB-MCI | 71  (15.01%) | 54  (13.37%) | 17  (24.64%) |  | 35  (17.24%) | 30  (17.75%) | 5  (14.71%) |  | 123  (68.33%) | 77  (63.11%) | 46  (79.31%) |  |
| **GDS** | 2.00 [0.00;3.00] | 2.00 [0.00;3.00] | 3.00 [1.00;5.00] | **<0.001** | 2.00 [1.00;3.00] | 1.00 [0.00;3.00] | 3.00 [2.00;4.75] | **<0.001** | 6.00 [3.00;7.00] | 6.00 [3.00;7.00] | 6.00 [3.00;7.00] | **0.809** |
| **STAI** | 61.0 [49.0;75.0] | 59.5 [48.0;73.0] | 70.0  [60.0;87.0] | **<0.001** | 60.0 [50.0;75.5] | 59.00 [50.00;74.00] | 67.50 [52.25;78.75] | **0.178** | 43.00 [42.00;45.00] | 43.00 [42.00;45.00] | 43.00 [41.00;45.00] | **0.783** |
| **SCOPA** | 10.00 [6.00;14.00] | 9.50 [6.00;13.00] | 12.00 [9.00;18.00] | **<0.001** | 10.00 [7.00;14.50] | 10.00 [7.00;14.00] | 13.50 [9.00;19.75] | **0.003** | 4.00 [3.00;6.00] | 4.00 [3.00;6.00] | 4.00 [2.00;6.00] | **0.595** |
| **Updrs I** | 6.0 [4.0;9.0] | 6.0  [3.0;9.0] | 7.0 [5.0;12.0] | **<0.001** | 6.0  [4.0;9.0] | 6.0 [4.0;9.0] | 8.50 [5.25;12.00] | **0.003** | 8.00 [5.00;11.00] | 8.00 [5.00;10.00] | 9.00 [6.00;11.75] | **0.053** |
| **Updrs II** | 6.00 [4.00;11.00] | 6.00 [3.00;9.00] | 12.00 [6.00;14.00] | **<0.001** | 6.00 [4.00;10.00] | 6.00 [3.00;9.00] | 10.50 [6.25;13.75] | **<0.001** | 5.00 [3.00;8.00] | 5.00 [3.00;7.00] | 6.00 [4.00;11.00] | **0.014** |
| **Updrs III** | 25.0 [20.0;32.0] | 24.5 [19.0;32.0] | 27.0 [21.0;34.0] | **0.044** | 25.0 [20.0;34.0] | 25.00 [20.00;34.00] | 26.00 [21.00;33.00] | **0.804** | 41.00 [29.00;51.00] | 41.00 [29.25;51.00] | 41.00 [29.25;53.75] | **0.759** |
| **ESS** | 5.00 [3.00;8.00] | 5.00 [3.00;6.00] | 12.00 [10.00;14.00] | **<0.001** | 5.00 [3.00;8.00] | 5.0 [3.0;7.0] | 11.0 [10.0;13.0] | **<0.001** | 5.00 [3.75;11.00] | 4.00 [2.00;5.00] | 14.00 [11.25;15.00] | **<0.001** |

PPMI: Parkinson Progression Markers Initiative; SPH-PD: Shenzhen People’s Hospital-Parkinson’s disease; EDS: excessive daytime sleepiness; RH: right-handed; LH: left-handed; MH: mixed-handed; BMI: Body mass index; DOMSIDE: dominant side of onset; PDTRTMNT: PD treatment status; MCI: mild cognitive impairment; GDS: Geriatric Depression Scale; STAI: State-Trait Anxiety Index; SCOPA: Scales for Outcomes in Parkinson’s Disease-Autonomic;UPDRS: Unified Parkinson’s Disease Rating Scale; ESS: Epworth Sleepiness Scale.
