# Supplementary material for: Machine learning–based prediction of excessive daytime sleepiness in patients with Parkinson’s disease: findings from the PPMI cohort with external validation
Source: Front Med (Lausanne). 2026 Jul 20;13:1894008. doi: 10.3389/fmed.2026.1894008 (PMC13431447; doi:10.3389/fmed.2026.1894008)
Supplement: Supplementary file 1 [file Data_Sheet_1.zip › Revised version of Supplementary materials/Supplementary Table S3.doc]

**Supplementary Table S3**. Univariate analysis of predictors for EDS in patients with PD in the training dataset.

| **Predictor variables** | ***β*** | ***SE*** | **OR 95% CI** | ***Z*** | ***P*** |
| --- | --- | --- | --- | --- | --- |
| UPDRS III | 0.03 | 0.01425 | 1.031 (1.002-1.06) | 2.117 | 0.034 |
| UPDRS II | 0.17 | 0.02794 | 1.185 (1.123-1.254) | 6.075 | <0.001 |
| UPDRS I | 0.106 | 0.02524 | 1.111 (1.058-1.168) | 4.182 | <0.001 |
| SCOPA | 0.078 | 0.01845 | 1.081 (1.043-1.121) | 4.2 | <0.001 |
| STAI | 0.028 | 0.00651 | 1.028 (1.015-1.041) | 4.252 | <0.001 |
| GDS | 0.201 | 0.04399 | 1.222 (1.121-1.334) | 4.562 | <0.001 |
| MCI | 0.751 | 0.31532 | 2.119 (1.118-3.874) | 2.381 | 0.017 |

EDS: excessive daytime sleepiness; MCI: mild cognitive impairment; GDS: Geriatric Depression Scale; STAI: State-Trait Anxiety Index; SCOPA: Scales for Outcomes in Parkinson’s Disease-Autonomic; UPDRS: Unified Parkinson’s Disease Rating Scale; PD: Parkinson’s disease.
