# Supplementary material for: Machine learning–based prediction of excessive daytime sleepiness in patients with Parkinson’s disease: findings from the PPMI cohort with external validation
Source: Front Med (Lausanne). 2026 Jul 20;13:1894008. doi: 10.3389/fmed.2026.1894008 (PMC13431447; doi:10.3389/fmed.2026.1894008)
Supplement: Supplementary file 1 [file Data_Sheet_1.zip › Revised version of Supplementary materials/Supplementary Table S4.doc]

**Supplementary Table S4.** Performance metrics in external validation cohorts.

| **Model** | **Accuracy** | **Balanced Accuracy** | **F1-score** | **PR-AUC** |
| --- | --- | --- | --- | --- |
| NB | 0.428 | 0.528 | 0.477 | 0.545 |
| LR | 0.678 | 0.595 | 0.42 | 0.545 |
| XGB | 0.683 | 0.549 | 0.26 | 0.545 |
| LGBM | 0.694 | 0.576 | 0.337 | 0.545 |

LR: logistic regression; XGB: extreme gradient boosting; NB: naive Bayes; LGBM: light gradient boosting machine.
